# Chromosome Instability, Aging and Brain Diseases

**DOI:** 10.3390/cells10051256

**Published:** 2021-05-19

**Authors:** Ivan Y. Iourov, Yuri B. Yurov, Svetlana G. Vorsanova, Sergei I. Kutsev

**Affiliations:** 1 Yurov’s Laboratory of Molecular Genetics and Cytogenomics of the Brain, Mental Health Research Center, 117152 Moscow, Russia; y_yurov@yahoo.com (Y.B.Y.); svorsanova@mail.ru (S.G.V.); 2 Laboratory of Molecular Cytogenetics of Neuropsychiatric Diseases, Veltischev Research and Clinical Institute for Pediatrics of the Pirogov Russian National Research Medical University, 125412 Moscow, Russia; 3 Department of Medical Biological Disciplines, Belgorod State University, 308015 Belgorod, Russia; 4 Research Centre for Medical Genetics, 115522 Moscow, Russia; kutsev@mail.ru

**Keywords:** aging, aneuploidy, brain diseases, chromosome, chromosome instability, genome instability, neurodegeneration

## Abstract

Chromosome instability (CIN) has been repeatedly associated with aging and progeroid phenotypes. Moreover, brain-specific CIN seems to be an important element of pathogenic cascades leading to neurodegeneration in late adulthood. Alternatively, CIN and aneuploidy (chromosomal loss/gain) syndromes exhibit accelerated aging phenotypes. Molecularly, cellular senescence, which seems to be mediated by CIN and aneuploidy, is likely to contribute to brain aging in health and disease. However, there is no consensus about the occurrence of CIN in the aging brain. As a result, the role of CIN/somatic aneuploidy in normal and pathological brain aging is a matter of debate. Still, taking into account the effects of CIN on cellular homeostasis, the possibility of involvement in brain aging is highly likely. More importantly, the CIN contribution to neuronal cell death may be responsible for neurodegeneration and the aging-related deterioration of the brain. The loss of CIN-affected neurons probably underlies the contradiction between reports addressing ontogenetic changes of karyotypes within the aged brain. In future studies, the combination of single-cell visualization and whole-genome techniques with systems biology methods would certainly define the intrinsic role of CIN in the aging of the normal and diseased brain.

## 1. Introduction

Sixty years ago, intercellular changes in chromosome numbers were demonstrated to be a possible cellular mechanism of human aging [1]. Later on, aging was systematically associated with the accumulation of aneuploid cells (i.e., cells exhibiting the loss/gain of whole chromosomes) and, occasionally, with chromosome instability (CIN) [2,3,4]. Furthermore, the rates of the latter were found to increase throughout ontogeny, as documented by studying the variability of cancer genomes [5,6]. Currently, somatic chromosomal mosaicism (mosaic aneuploidy) and CIN are suggested to contribute to aging processes in health and disease [7]. Still, the causes and consequences of CIN in aging post-mitotic tissues remain poorly understood.

Genome instability (including instability at the chromosomal/subchromosomal level) seems to be involved in the normal and pathogenic aging of the human brain [8,9]. CIN-associated diseases (i.e., CIN syndromes) exhibit progeroid phenotypes and/or cellular phenotypes hallmarking aging processes [10,11]. Significantly, CIN may represent a mechanism for neurodegeneration in diseases featured by accelerated aging [12]. Similarly, the brain of individuals suffering from Alzheimer’s disease (a late-onset neurodegenerative disease suggested to be linked somehow to pathological brain aging) is characterized by high rates of CIN [13], which mainly manifests as aneuploidy [14,15,16]. In total, somatic mutagenesis leading to CIN and chromosomal mosaicism may be considered an element of molecular and cellular pathways to normal and pathogenic aging mediating a variety of diseases [17]. If CIN is confined to the brain, one may expect a progressive neuropathological process resulting in a devastating neuropsychiatric illness. Among the latter, aging-related neurodegenerative diseases are the most common ones [7,15,18]. Accordingly, understanding the interplay between CIN and brain aging appears to be a key for unraveling the mechanisms of neurodegeneration and explaining brain deterioration in late life.

Here, we address ontogenetic aspects of CIN in light of brain aging with a special focus on neurodegenerative diseases. Heterogeneous data about CIN in the aging brain is considered in the context of forthcoming research dedicated to cytogenomic solutions to problems surrounding genome behavior during brain aging, pathways to age-related CIN, possible antiaging therapy, and the detection of the intrinsic rates of CIN in the healthy and diseased brain.

## 2. CIN in the Human Brain: An Ontogenetic View

CIN and mosaic aneuploidy are considered to play a role in brain development and functioning [7,19]. These types of genomic variations are more likely to possess a special meaning for the brain due to the organizational specificity of the central nervous system. Briefly, even a small proportion of genetically abnormal (neuronal) cells might affect brain functioning because of a multitude of intercellular connections (synapses) [14,20]. Taking into account the post-mitotic nature of the mammalian brain, the origins of brain-specific CIN are more likely to be developmental. The developing human brain (8–11 weeks) is shown to be significantly affected by CIN almost exclusively manifesting as aneuploidy. The proportions of abnormal cells may achieve 30–35% [21,22]. At the subchromosomal level, copy number variations (CNVs) below 1 Mb in size are found to underlie the genomic diversification of cells in the developing mammalian brain [23,24]. However, since large-scale intercellular genomic variations (e.g., aneuploidy) are exclusive at later developmental stages [24], an extensive decrease of CIN-affected cells is likely to exist. As reportedly noted, brain-specific developmental CIN significantly decreases to become less abundant in the postnatal brain through a sophisticated mechanism of regulating neural populations (e.g., aneuploidization followed by mitotic catastrophe) [3,17,19,25]. Postnatally, the result of these orchestrated changes could be traces of the presence of abnormal neural cells during prenatal development. Alternatively, a variety of phenomena (DNA replication stress, cellular senescence [26,27]) are able to favor the conservation of CIN rates or even promote CIN progression after birth throughout adulthood. When transformed into an appreciable population, cells affected by CIN/somatic mosaicism are able to become a source for morbidity and aging [28,29,30]. Moreover, alterations to a number of molecular/cellular pathways (DNA damage response; RNA somatic gene recombination; mTOR, PI3K-Akt, p53, PTEN, MAPK) predispose to CIN/mosaic aneuploidy in the diseased and aged brain [31,32,33]. To highlight the contribution of CIN to normal and pathological brain aging, it seems important to address aging-associated diseases characterized by brain malfunction (e.g., neurodegeneration) and brain-specific CIN.

## 3. CIN in the Diseased Brain: An Aging Perspective

During the last two decades, an appreciable amount of data on chromosomal variations (aneuploidy) and CIN directly affecting the brain was provided. Currently, it is suggested that several neurodevelopmental, psychiatric and neurodegenerative disorders may be associated with CIN and somatic chromosomal mosaicism confined to the brain or even to specific brain areas [12,14,16,18,19,20,33,34]. Brain tissue-specific chromosomal mosaicism and CIN are detectable in a significant proportion of cases of common brain diseases, including schizophrenia, autism/intellectual disability and Alzheimer’s disease [7,18,30,33]. However, CIN is a significantly more prominent biomarker of neurodegeneration (neurodegenerative diseases) when comparing intercellular chromosomal or (cyto)genomic variations between different types of brain disorders with a special emphasis on brain aging [12,13,14,15,16,34,35].

Neurodegenerative diseases (Alzheimer’s disease and common non-Alzheimer’s disease dementias) are systematically associated with a wide spectrum of genomic variations. Among others, these genomic changes may affect molecular (cellular) pathways of genome stability maintenance or protection against CIN (e.g., cell cycle regulation) [36,37]. The ability for neurons to enter erroneously into the cell cycle seems to underlie the formation of aneuploidy and other types of CIN in the diseased (Alzheimer’s disease) brain [9,34,35,38,39]. Actually, CIN/aneuploidy confined to affected brain areas has been determined as an important element of the pathogenic cascade in Alzheimer’s disease [40,41]. In addition, the Alzheimer’s disease brain has been shown to exhibit a variety of CIN types. Moreover, a number of parallels between Alzheimer’s disease and cancer directly related to cellular phenotypes and pathological processes leading to CIN/aneuploidy appear to exist [42]. More crucially, genes associated with Alzheimer’s disease (including genes mutated in rare familial cases) may induce chromosome missegregation and aneuploidy, leading to CIN in the diseased brain [40,43,44]. Brain-specific Alzheimer’s disease-associated aneuploidy/CIN more commonly involves chromosome 21 (note: *APP* (Amyloid Beta Precursor Protein) gene is located on chromosome 21) [14,16,44]. These findings correlate with long-standing observations concerning neurological and molecular parallels between Alzheimer’s disease and Down syndrome (trisomy of chromosome 21 or the presence of an extra chromosome 21 in the overwhelming majority of cells) [45]. Furthermore, X chromosome aneuploidy or X chromosome loss/monosomy [46] (i.e., CIN specifically affecting chromosome X [35]) has been found to hallmark the Alzheimer’s disease brain. It is of note that X chromosome loss is the most documented cytogenetic (chromosomal) biomarker of human aging [2,4]. CNVs, resulting from somatic recombination and selectively affecting the *APP* gene, have also been found to produce genomic or subchromosomal instability in the Alzheimer’s disease brain [47]. This type of genomic mosaicism is probably the result of the somatic gene recombination of mRNA/ncRNA [32]. Uncorrected DNA damage, which is able to initiate CIN, is also a biomarker of Alzheimer’s disease [48]. At the proteomic level, the abnormal functioning of cell cycle proteins is suggested to produce aneuploidy and other CIN types in post-mitotic neurons of the Alzheimer’s disease brain [49]. Finally, DNA replication stress appears to underlie CIN in the Alzheimer’s disease brain, allowing a theoretical link between two major hypotheses of the disease: the amyloid hypothesis and cell cycle hypothesis [50]. These data on genome/chromosome behavior in the Alzheimer’s disease brain have been commonly correlated with disease phenotype and peculiarities (e.g., sex differences) [44,45,51]. In summary, despite the debates concerning the contribution of brain aging to the pathophysiology of Alzheimer’s disease, one has to admit the involvement of CIN, which mediates aging in mitotic tissues and initiates from aging-related alterations to cellular homeostasis, in the pathogenic cascade.

It is noteworthy that Alzheimer’s disease is not the unique neurodegenerative disorder associated with aneuploidy and/or CIN. A CIN syndrome characterized by accelerated aging and neurodegeneration (ataxia-telangiectasia) exhibits cerebellar-specific CIN (chromosome breaks and rearrangements), which mediates neurodegeneration [12] and brain-specific aneuploidy [14]. Furthermore, the ataxia-telangiectasia brain picturesquely demonstrates region-specific effects of CIN. Thus, chromosome 14-specific instability (interphase chromosome breaks and additional rearranged chromosomes 14) confined to the cerebellum mediates neurodegeneration [12]. When analyzing regional genomic DNA content variation in different cortical areas of the Alzheimer’s disease brain, traces of a similar effect have been observed [34].

Lewy body diseases have been associated with aneuploidy in the diseased brain [52]. More strikingly, frontotemporal lobar degeneration caused by *MAPT* (microtubule-associated protein tau) mutations has been found to exhibit mitotic defects that lead to neuronal aneuploidy and apoptosis in the diseased brain [53]. Additionally, *MAPT* mutations cause CIN and introduce CNVs widely in the genome [54]. Interestingly, CNVs appreciably contribute to the most common aging-related neurodegenerative disorders [55]. Unfortunately, there are few studies dedicated to analyzing brain-specific CIN in other neurodegenerative conditions than Alzheimer’s disease. Still, genetic and (cyto)genomic analyses of the neurodegenerating brain have connected mutations in genes involved in safeguarding genome stability, CIN and molecular pathways in neurodegenerative diseases [33]. Similarly to Alzheimer’s disease [40,44], mitotic dysfunction and cell cycle errors are common features of neurodegenerative diseases, as a whole [56]. Recently, a theoretical model has allowed the determination of the difference between cancerous and neurodegenerative CIN. Neuronal cell death seems to be a key element of the pathogenic cascade initiating the progressive loss of cells affected by CIN [57]. Therefore, one can conclude that the culmination of brain-specific CIN accumulation is likely to be a starting point for the progressive clearance of neurons leading to neurodegeneration.

In addition to genome stability maintenance, cell cycle regulation and neuronal cell death, it is worth mentioning cellular senescence in the light of brain aging and neurodegenerative diseases. Cell senescence represents a homeostatic process characterized by sustained cell cycle arrest and a distinct cellular phenotype. It is able to make a contribution to a decrease in the regenerative potential and to alterations of tissue functioning. Cellular senescence is currently suggested to be involved in the pathophysiology of neurodegenerative diseases and brain aging [58,59,60]. Despite the lack of consensus on the role of cell senescence in Alzheimer’s disease pathology, it is accepted that the cellular senescence pathway is tightly connected to the neurodegenerative processes during brain aging [60]. The presence of senescent cells in a tissue hallmarks aging-related processes in health and disease [61]. Admittedly, interplay between CIN, pathways to CIN and cellular senescence would be a missing link in the pathophysiology of normal and abnormal brain aging.

## 4. CIN in the Aged Brain: The Shape of Things to Come

Focusing on cellular senescence has revealed the tight connection to cell cycle regulation, neuronal cell death and genome stability maintenance in the aged brain [61,62]. Furthermore, aging-related mechanisms for neurodegeneration have the potential to lead both to CIN and cellular senescence [63,64]. As systematically shown, cell senescence is linked to genome instability/CIN and neuronal cell death [31,65,66]. This link has been additionally demonstrated by a study that shows the inhibition of aging-associated CIN delaying cellular senescence [67]. Taking into account the dynamic nature of CIN and somatic mosaicism rates both in mitotic and post-mitotic cellular populations [68], one may suggest that cellular senescence arises from CIN in brain aging. This idea is further supported by a line of evidences for CIN manifesting as aneuploidy progressing in the aged brain [69,70,71]. It is of note that the traces of mitotic dysfunction hallmarking aging [72] have been found in the aged and diseased brain [9,25,34]. Moreover, the genetic-environmental interactions in the brain of individuals with aging-related diseases and CIN involve molecular pathways of programmed cell death and genome stability maintenance [73]. Thus, CIN affecting the aged brain explains a number of aging-related phenomena/processes (cellular senescence, mitotic machinery exhaustion, (neuronal) cell loss, proinflammatory response), which define tissular and cellular phenotypes specific for aging [3,29,59,74]. Chromosome-specific instability (e.g., chromosome 14-specific instability—ataxia-telangiectasia; chromosome 21-specific instability—Alzheimer’s disease) appears to represent another possible mechanism for the age-dependent malfunction of the brain. At this point, the involvement of X-chromosome-specific aneuploidy (X-chromosome-specific instability) may help to explain sex differences in the aging of the healthy and diseased (Alzheimer’s disease) brain. More precisely, increased rates of X chromosome loss (specific for female karyotypes) in the Alzheimer’s disease brain [46] might specifically contribute to the disease course in females [51,75]. X-chromosome-specific aneuploidy may not be a unique type of CIN contributing to sex differences in the Alzheimer’s disease brain [35]. Finally, DNA replication stress and DNA double-strand breaks are able to result in somatic CNVs affecting smaller genomic regions comparing to aneuploidy (i.e., whole chromosomes gains/losses) [50,76]. Since CNVs are able to contribute to common aging-related neurodegenerative diseases [55], it is probable that CIN generated by CNVs, which has the potential to progress and affect cellular homeostasis (for more details, see [77]), can also contribute to normal and pathological brain aging. In turn, this CIN type, being poorly compatible with the cellular lifespan, may lead to neuronal cell death.

**Figure 1:**
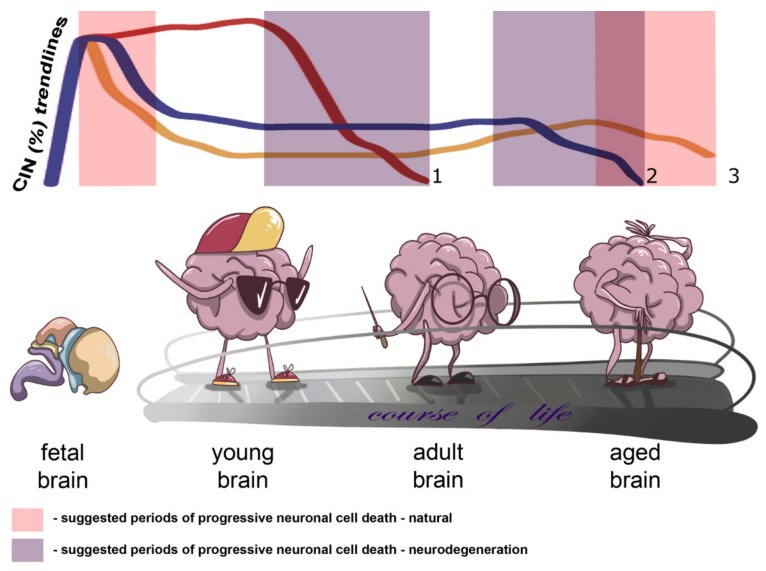
Schematic depiction of changes in chromosome instability CIN rates in the context of brain aging and neurodegeneration indicating trends of brain-specific CIN rates through ontogeny and/or the course of life and suggested periods of progressive neuronal cell death in health and disease (natural and neurodegeneration, respectively): 1 or reddish trendline—CIN trend for early onset neurodegenerative diseases with accelerated aging phenotypes, e.g., ataxia-telangiectasia; 2 or blueish trendline—CIN trend for late onset neurodegenerative diseases; 3 or yellowish trendline—natural CIN trend.

Onto(cyto)genetic views on the human brain allowed us to propose a kind of a model for neural genome behavior in connection to CIN and normal/pathological aging. On the one hand, brain-specific CIN producing senescence in neuronal cell populations may progress slowly throughout the adult life, whereas on the other hand CIN may become a trigger for progressive neuronal cell death in late ontogeny. While it certainly exists, a ‘point of no return’ for CIN-mediated neuronal cell death has not been comprehensively described. We believe that either critically adverse effects on cellular homeostasis produced by CIN or CIN-initiated programmed cell death (e.g., mitotic catastrophe) may be involved. In aging-related brain diseases, the process of losing CIN-affected cells is likely to be more dramatic (fast) and to begin earlier than in presumably unaffected individuals. The differences between pathological and normal brain aging may result from variable degrees of alterations to the aforementioned pathways. An additional source for the difference might be chromosome breakages or CNVs produced by CIN, which lead to improper functioning (activation/inactivation) of different spectra of genes in the affected neuronal cell population. However, such an effect has not as yet been empirically addressed. In total, CIN is likely to be involved in cell number regulation during early and late ontogeny or in neurodegeneration. Figure 1 demonstrates the essence of our assumption concerning the changes in the rates of brain-specific CIN by indicating the trendlines of brain-specific CIN rates through ontogeny and highlighting suggested periods of progressive neuronal cell death in health and disease.

The dynamic nature of CIN and somatic chromosomal mosaicism has been recently suggested as a target for the exogenous control of the rates. The control is an opportunity for the diminishment of CIN rates and, consequently, for tissular rejuvenation, increasing the lifespan and slowing down disease progression [68]. In the available literature, suggestions concerning the realization of this idea have been already proposed. It is more likely that genetic-environmental interactions may help to inhibit CIN or eliminate affected cells at early stages of aging and/or disease [71,73]. A candidate process for these interactions would be mitotic/cell cycle regulation, programmed cell death and cellular senescence [49,53,59,60,64]. For instance, the analysis of DNA damage response in neurons shows the possibility of switching between programmed cell death (apoptosis) and a pseudo-stationary cellular state (senescence-like state) [31]. Currently, following targets for therapeutic interventions in aging and neurodegeneration has been empirically defined: DNA double-strand breaks [76], aging-associated CIN (the inhibition by small-molecule enhancement of microtubule-depolymerizing kinesin-13 activity delays cellular senescence) [67], somatic gene recombination of mRNA/ncRNA [32], and the deterioration of nuclear morphology and architecture mediated by cell senescence [78]. As was recently noted, ‘the aging genome’ is to be protected for successful antiaging therapies [79]. It is more probable that the basis of these therapies is a pathway-based analysis providing an opportunity to define molecular interventions towards longevity in health and disease [80,81]. To perform such an analysis, a closer look at the whole set of genomic changes produced by CIN is required [82,83]. Additionally, determining the susceptibility/tolerance to brain-specific CIN using a whole-genome scan and systems biology techniques may be useful for preventing/inhibiting CIN progression and related processes [68,73,82]. Summarizing neurogenomic and molecular neurocytogenetic data leads one to conclude that CIN-related pathways are promising targets for antiaging therapy or brain rejuvenation and for therapeutic interventions in neurodegenerative diseases. Therefore, possible therapeutic strategies are likely to be based on molecular cytogenetic (cytogenomic), whole-genome and systems biology analyses focused on the interplay between the genetic and environmental causes of CIN in the brain.

To this end, we have to mention that data on aneuploidy that was found to be increased in the aged brain by visualization techniques [69,70,71] do not conform to data obtained by studies using single-cell whole-DNA-fraction analyses, which have shown a lack of aneuploid cells in the aged human neocortex [84]. To solve this problem, we can propose the use of a previously described workflow proposed for the single-cell analysis of cellular genomes in the brain. The workflow combines molecular cytogenetic (visualization), whole-genome (single-cell and multiple-cell analysis of DNA fractions) and systems biology (bioinformatics) techniques (for more details, see [85]). Alternatively, one can suppose that CIN-affected neuron loss via neuronal cell death explains the contradiction between reports addressing ontogenetic changes in karyotypes within the aged brain. In other words, a number of (cyto)genomic studies address the aged brain at the ontogenetic stages when CIN-affected neurons have been already lost. In the future, single-cell analysis using the workflow mentioned below may help to uncover the basis of the discrepancies between studies on chromosome complements in the aged brain.

## 5. Concluding Remarks

In 1990, about 300 hypotheses for aging were described in the available literature [86]. Since then, the list has been extended by those dealing with mitotic machinery exhaustion, genome instability/CIN in post-mitotic tissues, programmed death of post-mitotic cells, DNA replication stress, DNA damage response, DNA repair, DNA double-strand breaks, etc. [16,25,48,50,57,65,66,75]. Nonetheless, a generalized theory encompassing the majority of original ideas expressed in these hypotheses does not exist. To simplify the modeling of aging, nine tentative hallmarks were introduced. These are: genomic instability (1), telomere attrition (2), epigenetic alterations (3), loss of proteostasis (4), deregulated nutrient sensing (5), mitochondrial dysfunction (6), cellular senescence (7), stem cell exhaustion (8) and altered intercellular communication (9) [66]. As one may see, at least four of these hallmarks (1–3 and 7) are related to CIN. More importantly, genomic instability (genomic instability and CIN interfere with each other) and cellular senescence are involved in the pathogenesis of aging-related (neurodegenerative) brain diseases. Furthermore, it appears that the aging-related deterioration of the brain is likely to be mediated by a cascade that involves CIN, cell senescence and neuronal cell death. We suppose that brain-specific CIN rates are able to increase slowly throughout adulthood. However, during later ontogenetic periods, CIN-affected neurons are likely to be cleared by neuronal cell death. As a result, the number of neurons in the aged brain may be significantly diminished. In neurodegenerative diseases, these processes are more dramatic and are, thereby, more apparent than in natural brain aging. Thus, CIN and cell senescence pathways might be a target for antiaging therapy (brain rejuvenation) and therapeutic interventions in debilitating neurodegenerative disorders. Certainly, a model based on the sophisticated interplay between cell cycle regulation, DNA reparation, CIN and cellular senescence should not be considered as the ultimate one. We suggest that a synthesis of the rationales obtained by aging studies from different areas of biomedicine is the most promising way to understand human aging.

Future research dedicated to the genetic and (cyto)genomic aspects of brain aging has to combine visualization, whole-genome (single-cell/multiple-cell analysis of DNA fractions) and systems biology (bioinformatics) techniques for the determination of intrinsic CIN/aneuploidy rates and CIN-associated cellular phenotypes (e.g., cellular senescence). Once acquired, this knowledge can be used to develop effective strategies for brain rejuvenation and neurodegeneration treatment.

## Data Availability

Not applicable.
